# Co-delivery of orthodontic treatment: Perceptions of supervising clinicians and orthodontic therapists

**DOI:** 10.1177/14653125251391368

**Published:** 2025-11-15

**Authors:** Jonathan D Shelswell, Louise A Belfield, Simon J Littlewood, Sophy K Barber

**Affiliations:** 1 Department of Orthodontics, University of Leeds, Leeds, UK; 2 Head of assessment, Brunel Medical School, Brunel University London, London, UK; 3 Department of Orthodontics, Bradford Teaching Hospital NHS Foundation Trust, Bradford, UK; 4 Department of Orthodontics, University of Leeds, Leeds, UK

**Keywords:** economics of orthodontic delivery systems, health services and quality of life aspects, quality of life and orthodontics

## Abstract

**Objectives:**

To establish how orthodontics is currently co-delivered by orthodontic therapists (OTs) and supervising clinicians (SCs), and to explore both sets of clinicians’ perceptions of these working arrangements.

**Design and setting:**

Cross-sectional survey using an online questionnaire.

**Participants:**

General Dental Council (GDC)-registered OTs and specialist or non-specialist dentists who supervise OTs and work in the UK.

**Methods:**

A link to the online questionnaire was emailed to all members of the British Orthodontic Society and Orthodontic National Group and was posted in two Facebook groups. Reminder emails and Facebook posts were sent.

**Results:**

A total of 161 responses were received from 89 SCs and 72 OTs. Most worked in primary care as their main clinical role. Most OTs in primary care provided a mix of NHS and private care. Appointments with OTs were most likely to be supervised every other visit, with more frequent supervision reported by SCs, and by clinicians in secondary care. Remote supervision of some kind was reported by 63% of OTs. Different barriers and enablers to effective working practices were suggested by OTs and SCs. OTs reported improved patient satisfaction as the main consequence of their utilisation in the orthodontic workforce while SCs described improved clinical efficiency.

**Conclusions:**

OTs reported improved patient satisfaction as the main consequence of their utilisation, whereas SCs described improved clinical efficiency. Some OTs felt that SCs should be more readily available and that OTs should have more autonomy. SCs would prefer more time to supervise and provide prescriptions.

## Plain language summary

How do orthodontists and orthodontic therapists work together in the UK? Views from both groups

Orthodontic treatment, using braces, is often delivered by a team that includes orthodontists or dentists (supervising clinicians) and orthodontic therapists. This study explored how these two groups work together and what they think about their working relationships. An online questionnaire was shared with UK-based orthodontists, dentists and orthodontic therapists. A total of 161 people responded: 89 supervising clinicians and 72 orthodontic therapists. Most of them worked mainly in primary care (for example, high street dental practices) and treated both NHS and private patients. The study found that orthodontic therapists are usually supervised by a dentist or orthodontist at every other appointment. Clinicians working in hospital settings tended to supervise therapists more often. Some therapists also said they worked under ‘remote supervision’, meaning the supervising clinician was not always present during the appointment. Both groups described different challenges and advantages in working together. Orthodontic therapists said their work improved patient satisfaction, helping patients feel happier with their treatment experience. Supervising clinicians highlighted that working with orthodontic therapists made clinical processes more efficient. However, some orthodontic therapists felt that supervising clinicians should be more available to support them during appointments, and that therapists should be allowed to work more independently. On the other hand, supervising clinicians said they would like more time for supervision and for providing detailed treatment plans. This study shows that co-delivery of orthodontic care can benefit patients and practices, but both orthodontic therapists and supervising clinicians see areas where working relationships and support could be improved.

## Introduction

The first orthodontic therapists (OTs) registered with the General Dental Council (GDC) in 2007, after a significant period of campaigning by orthodontists in the UK (The British Orthodontic Society Archive and Museum Committee, 2011). Orthodontic ‘auxiliaries’ had been in use for some time in Europe, the USA, Canada and Australia before the UK to address the lack of orthodontic specialists (Blau, 1973; Graebner, 1975; Moss, 1993; O’Brien and Shaw, 1988; Pollard, 2000). The introduction of OTs in the UK was, in part, to address unmet orthodontic need and reduce regional variations in the distribution of orthodontists and orthodontic care (O’Brien et al., 1989). The GDC currently recognises eight OT programmes providing a Diploma in Orthodontic Therapy after at least 45 weeks of full-time training (General Dental Council, 2019).

OTs are permitted to undertake reversible orthodontic procedures under the prescription of a dentist, as shown in Table 1 (General Dental Council, 2013). Since their introduction in the UK, there has been discussion about the appropriate level of supervision of OTs (Hodge and Parkin, 2015). The British Orthodontic Society (BOS) published its first position statement on OT supervision in 2011, and was most recently updated in 2023 (British Orthodontic Society and Orthodontic National Group, 2023). They make clear that the supervising clinician should see the patient at least every other visit.

**Table 1.** The General Dental Council scope of practice for orthodontic therapists.

Permitted to undertake if trained, competent and indemnified:	Additional skills with further training:
Clean and prepare tooth surfaces ready for orthodontic treatment.	Applying fluoride varnish to the prescription of a dentist.
Identify, select, use and maintain appropriate instruments.	Repairing the acrylic component part of orthodontic appliances.
Insert passive removable orthodontic appliances.	Measuring and recording plaque indices.
Insert removable appliances activated or adjusted by a dentist.	Removing sutures after the wound has been checked by a dentist.
Remove fixed appliances, orthodontic adhesives and cement.	
Identify, select, prepare and place auxiliaries.	
Take impressions.	
Pour, cast and trim study models.	
Make a patient’s orthodontic appliance safe in the absence of a dentist.	
Fit orthodontic headgear.	
Fit orthodontic facebows that have been adjusted by a dentist.	
Take occlusal records including orthognathic facebow readings.	
Take intra- and extraoral photographs.	
Place brackets and bands.	
Prepare, insert, adjust and remove arch wires previously prescribed or, where necessary, activated by a dentist.	
Give advice on appliance care and oral health instruction.	
Fit tooth separators.	
Fit bonded retainers.	
Carry out IOTN screening either under the direction of a dentist or direct to patients.	
Make appropriate referrals to other healthcare professionals.	
Keep full, accurate and contemporaneous patient records.	
Give appropriate patient advice.	

IOTN, Index of Orthodontic Treatment Need.

The working practices of OTs were reported in 2018 through surveys of 211 OTs (Ahmed et al., 2018) and 747 orthodontists (Dugdale et al., 2018). At this time, OTs performed most clinical procedures and OTs usually performed clinical procedures ‘unsupervised and from a written prescription’ (Ahmed et al., 2018). Most (75%) orthodontists felt that the introduction of OTs had led to an increase in clinical output (Dugdale et al., 2018). Using the Peer Assessment Rating, Rooney et al. (2016) found no clinically important differences in occlusal outcomes before and after the introduction of OTs in primary and secondary care settings. This suggests the quality of orthodontic treatment has not been negatively impacted by the delivery of treatment by OTs.

However, the landscape of orthodontic practice has changed considerably in the years since the publication of this research, and the number of OT registrants has increased from 16 in 2007, to 364 in May 2014 (Hodge and Parkin, 2015), to 1015 in October 2023 (General Dental Council, 2023a). Furthermore, there is limited research that examines how the introduction of OTs has affected the provision of orthodontics. This study will report the perceptions of OTs and supervising clinicians (SCs) about their contribution to patient care, as well as the barriers and enablers to effective co-delivery of treatment. The term supervising clinicians has been used here to include both specialist orthodontists and non-specialist dentists who supervise OTs.

## Aims

The aims of the study were as follows: (1) to evaluate the delivery of orthodontic treatment by OTs and SCs (specialist orthodontists or dentists); and (2) to explore the perceptions of OTs and SCs about the delivery of orthodontic treatment.

## Study design

The study design comprised a cross-sectional survey conducted via an online questionnaire. Ethical approval was awarded by the University of Leeds Dental Research Ethics Committee (1 March 2023).

## Participants

The participants for the study were GDC-registered OTs and dentists and orthodontists who supervise OTs working in the UK.

## Methods

### Questionnaire development and design

The structure and content of the questionnaire was initially developed within the research team, before being refined with stakeholder input. No formal psychometric validation was undertaken. The first stakeholder meeting took place with two orthodontists and the second with four OTs. An initial questionnaire was developed then piloted by observing two OTs and three orthodontists completing the questionnaire, with immediate verbal feedback provided. Participants in the stakeholder meetings and testing were from a range of clinical roles in the NHS and private sector, working in primary and secondary care, with most having more than 10 years of experience since completion of specialist training or diploma.

The questionnaire consisted of four sections:

1. Respondent demographics
2. Working arrangements
3. Treatment delivery: appointment times, supervision levels and the actual procedures routinely completed by OTs
4. Outcomes: perceptions of the contribution of OTs to patient care, and whether the skill set and competence of OTs is most efficiently used

The final questionnaire was programmed into OnlineSurveys. A copy of the full questionnaire is included in the supplementary material.

### Recruitment

SCs were identified and invited using the entire BOS emailing list, and OTs through the Orthodontic National Group (ONG). Social media was also used for recruitment using the ‘Orthodontic Therapist Network UK’ and ‘Orthodontic Mastery Group’ Facebook groups. Participants self-enrolled, and consent to participate was implied by completing the questionnaire; a formal digital signed consent process was not undertaken. Participants working in more than one setting were given the option of repeating the questionnaire to report their different working arrangements.

### Data collection

Participants were able to access the online questionnaire through a link in the email or social media post. OnlineSurveys collected the responses anonymously and automatically collated the data.

### Data analysis

Quantitative data were reported using summary statistics, including response rates, frequencies, measures of central tendency and dispersion, and charts including histograms and bar charts produced in Microsoft Excel. Where questions did not have a 100% response rate, percentages were calculated using the total number of respondents who answered the specific question. Free text responses were categorised using a simple form of content analysis: comments were collated and responses compared between SCs and OTs by identifying common ideas or repeated ‘topics’. Categorisation was undertaken by a single researcher without independent verification or formal reliability testing, which, while providing a pragmatic approach, is recognised as a limitation.

## Results

Data collection was undertaken between 30 March and 30 June 23. A total of 161 responses were received from 89 SCs and 72 OTs. The response rate is difficult to quantify due to the varied methods of recruitment, but if the total sample size is taken as the number of recipients of the email invitations sent to the BOS and ONG mailing lists, this would indicate a response rate of approximately 6% for SCs (89/1483) and 35% for OTs (72/207), with a combined response rate of 9.5%.

### Respondent characteristics

For both OTs and SCs, the largest proportion of respondents reported that their main clinical role was in primary care. The number of non-specialist dentists supervising OTs was small, making up only 7.9% (n = 7) of all SCs, and most worked in dental hospitals (n = 4). Only 7 (4.3%) participants opted to complete the questionnaire for a second time from a different workplace perspective: two OTs in primary care, four orthodontists in primary care and one orthodontist in a district general hospital. These were treated as new responses when calculating statistics to give a total possible number of responses of 168 between 94 SCs and 74 OTs. For respondents who provided their level of experience (Table 2), the largest proportion had been an orthodontist for more than 20 years (37%, n = 24) and 11% (n = 7) had less than 5 years of experience as a specialist. Among the 50 OTs to provide this data, 20% (n = 10) had less than 5 years of experience, 34% (n = 17) had 5–10 years and 46% (n = 23) had 10–15 years of experience.

**Table 2.** Years of experience and clinical role of participants.

Respondents’ experience (years)	SC (n = 65)	OT (n = 50)
<5	7 (11)	10 (20)
5–10	9 (14)	17 (34)
10–15	9 (14)	23 (46)
15–20	16 (25)	
>20	24 (37)	
Clinical role	SCs (n = 91)	OTs (n = 56)
Primary care only	46 (51)	34 (61)
Secondary care only	17 (19)	8 (14)
Primary and secondary care	28 (31)	14 (25)

Values are given as n (%).

OT, orthodontic therapist; SC, supervising clinician. At the time of data collection, OTs had been established in the UK for no more than 15 years.

### Working arrangements

The split between working in primary and secondary care was broadly similar between SCs and OTs, with just over half working solely in primary care as their main clinical role (Table 2). Most SCs (n = 42, 71%) and orthodontic therapists (n = 44, 76%) worked in mixed NHS and private settings, with a median NHS:private split of 85:15; smaller proportions worked exclusively in either NHS or private practice (Table 3).

**Table 3.** The split between NHS, private and mixed practice for SCs and OTs whose ‘main clinical role’ was in primary care.

Role	100% NHS	100% Private	Mixed	Median NHS:private split for mixed
SC (n = 59)	11 (19)	6 (10)	42 (71)	85:15
OT (n = 58)	5 (9)	9 (16)	44 (76)	85:15

Values are given as n (%).

NHS, National Health Service; OT, orthodontic therapist; SC, supervising clinician.

The median (range) of the standard appointment lengths for various common scenarios for SCs and OTs is provided in Table 4. Broadly, there is little difference between the groups, with OTs reporting slightly shorter appointments for adjusting fixed appliances and placement of aligner attachments.

**Table 4.** Appointment lengths for SCs and OTs.

Procedure	Appointment length (min)	
	SC	OT
New patient assessment	20 (5–30)	
Records	20 (10–30)	20 (5–45)
Treatment planning/consent	20 (5–30)	20 (5–45)
Placement of dual-arch fixed appliances	45 (15–60)	45 (20–60)
Adjusting fixed appliances	20 (10–30)	15 (10–30)
Removal of dual-arch fixed appliances	30 (10–60)	30 (15–60)
Aligner check (without attachment placement/IPR)	15 (5–30)	15 (10–30)
Placement of aligner attachments	40 (15–60)	30 (15–60)

Values are given as median (range).

IPR, interproximal reduction; OT, orthodontic therapist; SC, supervising clinician. OTs do not undertake comprehensive new patient assessments and were not asked for this appointment length.

Of the 74 OTs who provided employment information, 65 (88%) were employed and 9 (12%) were self-employed. When asked whether they felt their salary appropriately reflected their contribution to patient care, 35% felt it did but 57% felt it did not, with no clear difference between those who were employed or self-employed.

### Supervision

**Figure 1.**
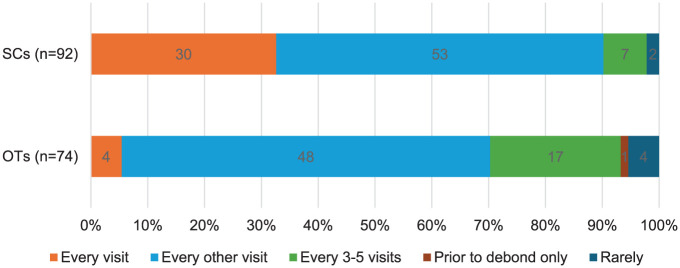
A comparison between the frequency of supervision reported by orthodontic therapists and supervising clinicians working across primary and secondary care.

Out of the total 166 responses, 101 (61%) reported the SC seeing the patient ‘every other visit’, 34 (20%) ‘every visit’, 24 (14%) ‘every 3–5 visits’, 6 (4%) ‘rarely’ and 1 (1%) ‘prior to debond only’. When comparing the responses from SCs and OTs, a similar proportion in each group selected ‘every other visit’; however, 30 (33%) SCs reported supervising patients ‘every visit’ compared to 4 (5%) OTs (Figure 1). The number of SCs who reported seeing patients ‘every 3–5 visits’ was 7 (8%), whereas OTs reported this number as 17 (23%). All OTs in secondary care reported supervision at least ‘every other visit’, compared to 39 (65%) OTs in primary care. After combining the ‘prior to debond only’ and ‘rarely’ categories due to small counts, a chi-square test confirmed a statistically significant difference between SCs and OTs in the reported frequency of supervision (χ² = 23.9; *P* < 0.001).

There was general agreement about supervision ratios between OTs and SCs. The median response for a ‘sensible’ number of OTs to be supervised if the SC was treating their own patients concurrently was two OTs. If the SC was not treating their own patients, the median ideal number to supervise was four OTs. When discussing their actual supervision arrangements, SCs reported that the median number of OTs routinely supervised by a single SC was two, whereas OTs reported this to be three.

The majority of SCs (73%) reported that they do not supervise remotely. However, more than half of OTs (63%) reported being supervised remotely at least sometimes. Most SC comments (n = 17) and some OT comments (n = 9) specifically described that this was not undertaken routinely. A smaller number of comments from SCs and OTs suggested that remote monitoring was more routinely undertaken in their clinical setting, with six comments mentioning the use of DentalMonitoring™.

### Scope of practice

**Figure 2.**
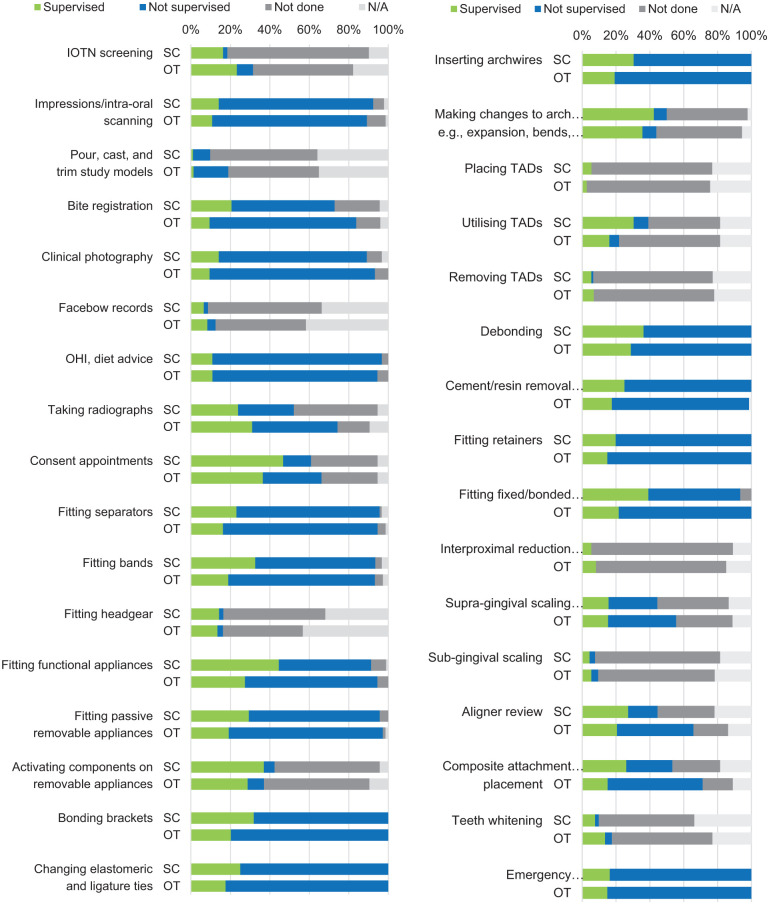
Procedures performed by OTs, as reported by supervising clinicians and OTs. OT, orthodontic therapist.

The procedures performed by OTs are summarised in Figure 2. When asked whether the skills of OTs are being appropriately utilised, a majority of SC (n = 83, 90%) and OT (n = 55, 74%) participants responded ‘Yes’. A chi-square test indicated a statistically significant difference between SCs and OTs (χ² = 7.3; *P* = 0.007), with a higher proportion of OTs (26%; n = 19) than SCs (10%; n = 9) reporting that their skills were not being fully utilised or selecting ‘Other’.

‘*Mostly but there are some erroneous rules. Like not being able to activate a URA*.’ [SC in primary care with 21 years of experience]

‘*I feel that we should be able to place simple activation in archwires, i.e. to coordinate a wax bite, to expand or constrict archwires or to sweep in a simple reverse curve*.’ [OT in primary care with 13 years of experience]

Two comments from experienced OTs pointed to a more limited utilisation due to the constraints of the current scope of practice:

‘*As a therapist who has been qualified for 15 years, I feel very restricted. There should be opportunities to increase scope*.’ [OT in primary care with 15 years of experience]

When asked directly whether OTs should be able to perform other clinical procedures as part of their scope of practice, 69 (75%) SCs responded ‘No’, whereas 50 (68%) OTs responded ‘Yes’. Free text comments left by SCs (n = 24) and OTs (n = 49) indicated which additional procedures OTs felt they should be able to perform (Table 5).

‘*I think that OTs. . . should be able to place small bends in archwires <0.75* *mm rather than repositioning the bracket as it has the same clinical effect as repositioning and has a very low risk of harm. . .*’ [SC in primary care with 26 years of experience]

‘*Wire bending but checked by orthodontist before placing it*.’ [SC in primary care with 6 years of experience]

**Table 5.** Suggestions for additional clinical procedures that should be included in the scope of practice of OTs.

Procedures	SC	OT
Interproximal reduction	9 (10)	19 (26)
Archwire bends	7 (8)	13 (18)
Teeth whitening	3 (3)	13 (18)
Activating removable appliances	5 (5)	10 (14)
Composite bonding	0 (0)	5 (7)
Quadhelix expansion	0 (0)	3 (4)
Incisal edge enameloplasty	0 (0)	3 (4)
Temporary restoration placement	0 (0)	1 (1)
Treatment planning	0 (0)	1 (1)
Mouthguards	0 (0)	1 (1)
Prescribe fluoride toothpaste/mouthwash	0 (0)	1 (1)
Prescribing radiographs	0 (0)	1 (1)
New patient assessment	0 (0)	1 (1)
PAR scoring	1 (1)	0 (0)
Subgingival scaling	1 (1)	0 (0)

Values are given as n (%).

IPR, interproximal reduction; OT, orthodontic therapist; SC, supervising clinician.

The majority of SCs and OTs agreed that OTs should not be able to make more decisions about treatment; however, more OTs (n = 27, 37%) than SCs (n = 7, 8%) felt OTs should have more decision-making capacity.

‘*I would like to be able to make decision within an already written treatment plan. For example, early wire changes whilst aligning*.’ [OT in primary care with 9 years of experience]

### Working relationships

Comments about the perceived effects of working with OTs on patient care and treatment efficiency were largely positive (Table 6), with SCs commenting on improved appointment availability and efficiency, and OTs highlighting improved outcomes and rapport with patients. Individual responses from SCs also reported improved job satisfaction as a supervisor. Although not directly related to the patient care and treatment efficiency, six OT comments reported a good relationship with their SC and three mentioned feeling valued:

‘*I am currently very satisfied with my clinical routine. I feel I am an appreciated member of the team who produces good treatment outcomes and patient satisfaction*.’ [OT in secondary care with 10 years of experience]

**Table 6.** Perceived benefits of OTs on patient care and treatment efficiency.

Topic	Number of times topic included in comments	
	SC (n = 82)	OT (n = 65)
More appointment availability	35	4
More patients seen	14	0
Good rapport with patients	11	0
More efficient	11	0
Good quality of care	9	7
Improved patient satisfaction	8	8
Good outcomes	7	12
Better use of skill mix	4	0
Continuity of care	4	2
Reduced treatment duration	2	0
Better delegation	2	0
Improved outcomes	2	0

OT, orthodontic therapist; SC, supervising clinician.

A smaller number of negative sentiments were also identified. Six OTs reported that their contribution was hindered by poor appointment availability and appointments that are too short:

‘*I don’t feel that it is more efficient seeing us as we don’t get a prescription given. The orthodontist still has to see the patient as well and can sometimes take up to 40 minutes for the supervisor to come and tell us what they want us to do*. . .’ [OT in secondary care with 8 years of experience]

Only two negative topics were mentioned more than once in SC comments, increased treatment duration (n = 6) and poorer outcomes (n = 4).

‘*More visits as OT generally less confident to progress treatment*.’ [SC in primary care with 32 years of experience]

The impact of working with orthodontic therapists on the clinical practice of supervising clinicians was reported in free text comments. Positive topics were identified 103 times in the comments, highlighting generally increased efficiency allowing more patients to be seen (n = 49). Improvements for patients were suggested through reported increased appointment availability (n = 8), reduced waiting times (n = 4), better service (n = 1) and improved quality of care (n = 1). Reported benefits for clinicians were lower levels of stress (n = 25), more enjoyable practice (n = 4), greater profitability (n = 3), greater delegation (n = 2) and the ability for SCs to focus more on complex care (n = 6).

Negative impacts on clinical practice were reported less frequently (n = 37), and a small number (n = 3) reported poorer quality of patient care. For clinicians, these focused on an increased administrative burden (n = 2), greater levels of organisation (n = 2), less wet-fingered dentistry (n = 2) and increased responsibility (n = 4). Interestingly, an almost equal number reported that working with OTs made their practice more stressful (n = 27) as those who said it made their work less stressful (n = 25). Many comments explained why and when it is more stressful, particularly when concurrently seeing their own patients while supervising:

‘*I do sometimes see my own patients with supervision responsibilities which does increase stress levels but when solely supervising, I feel working with OTs (especially with prescriptions) is incredibly effective*.’ [SC in secondary care with 8 years of experience]

‘*More stressful if OT is very busy, or if supervising many OTs with own busy list*.’ [SC in primary care with 4 years of experience]

Participants were asked about the facilitators to a good working relationship between SCs and OTs. Communication was the most important skill identified in the comments (SC = 55%, OT = 53%).

‘. . .*We are usually available in an adjoining surgery, or our offices, as well as written prescriptions in the notes. Face to face, email, and messages within notes all work well for communication*.’ [SC in secondary care with 21 years of experience]

‘*Excellent communication, discussing with rather than dictating to so I am always thinking and learning*.’ [OT in primary care]

Other positive interpersonal skills facilitating a good working relationship included professional rapport (SC = 13%, OT = 0%), teamwork (SC = 6%, OT = 6%) and approachability (SC = 5%, OT = 3%). The empowerment of OTs (SC = 4%, OT = 19%) and trust/respect (SC = 4%, OT = 19%) were also important to OTs especially.

Several working practices were also identified as enablers to a positive working relationship. Supervision was the most important aspect for both groups (SC = 25%, OT = 25%), but having defined roles (SC = 8%, OT = 8%), providing teaching and feedback (SC = 8%, OT = 0%), the use of a standard operating procedure (SC = 4%, OT = 0%), SC availability (SC = 0%, OT = 13%) and quality of prescriptions (SC = 1%, OT = 5%) were also mentioned.

Several comments described the benefits of training existing members of staff as OTs, and working with OTs who were trained by the same SC. Empowering OTs was a common perspective in OT responses and was also mentioned in three SC comments:

‘*I hope that we work well as a team. My aim is to ensure that my OT wants to come to work to see the patients but to also enjoy her day and not feel part of a machine churning out stuff – I feel I do more teaching in this setting as we are just two of us together*.’ [SC in primary care with 26 years of experience]

When asked what could be improved, the most frequent responses regarding supervision were from OTs, highlighting a need for increased availability of the SC and to not keep the OT waiting when they are with a patient:

‘. . . *he’s very busy so can’t see patients when they are in, so we take photos and he leaves a note for next visit prescription. I feel this prolongs treatment time for the patient*.’ [OT in primary care with 14 years of experience]

Having more time to supervise was the most frequently mentioned topic among the SC comments. To some extent, this overlapped with having time for feedback, the pressures of busy clinical practice and being able to have longer appointments:

‘*More time to explain to therapist your mechanics & better prescriptions for bond up and adjustment prescription for next visit*.’ [SC in primary care with 19 years of experience]

‘*Being less busy yourself*!’ [SC in primary care with 16 years of experience]

A desire for increased autonomy, or expansion of the scope of practice, was highlighted in eight OT comments. Four procedures were mentioned more than once by SCs: IPR, archwire bends, tooth whitening and the activation of removable appliances. Some OTs additionally reported incisal edge enameloplasty, composite bonding and activating quadhelices:

‘*Allowing OTs to perform IPR would improve the workflow*.’ [OT in primary care with 7 years of experience]

‘*Give a little more responsibility in early archwires with straight forward cases, knowing that I would seek advice if I felt necessary*.’ [OT in primary care with 13 years of experience]

## Discussion

### Summary

The findings of this survey have shown that OTs are largely valued and are perceived to add value to the orthodontic workforce. SCs widely reported the increased clinical efficiency when working with OTs, at times justifying the higher working stress of concurrently treating patients and supervising, whereas OTs reported improved patient satisfaction. Interestingly the appointment lengths for common procedures were almost identical, at times shorter with OTs. SCs may choose to see more complex cases themselves and may be factoring the additional time demand of supervision into their appointment lengths or it may simply be the case that there is a limit to how quickly certain procedures can be completed.

Even if utilising OTs does not save time over a patient’s overall treatment, the sentiment among SCs in this study that it increases efficiency and access to care is clear, which has been suggested in the literature (Hodge et al., 2015). Despite this, most OTs did not feel their remuneration reflected their contribution to patient care. Although research has been published regarding the working practices and career aspirations of OTs (Onabolu et al., 2018), a major strength of the present survey is the updated knowledge around the present employment, utilisation and supervision of OTs.

Evaluation of the perceptions of both SCs and OTs has demonstrated interesting and important differences in the perception of supervision and what does and does not work in the relationship. Although both groups of clinicians reported that good supervision and communication enabled a positive working relationship, the time pressure of clinical practice encapsulates the barriers identified; SCs would prefer to have more time to supervise, whereas OTs wanted more availability of SCs and to not have to wait for a prescription with a patient in the chair. There has been no research to date that demonstrates the ideal balance of available supervision time per OT appointment to optimise supervision.

The remote supervision of OTs has been reported for the first time, possibly as a result of the COVID-19 pandemic. The results of two early single-operator, retrospective cohort studies have suggested that using DentalMonitoring™ for patients undergoing clear aligner treatment leads to a reduced number of overall appointments (Hansa et al., 2020, 2021). However, both studies have a high risk of bias, do not involve OTs or their supervision, and only show a significant change in the overall number of appointments.

### Limitations

Although surveys have the advantage of reaching a large number of participants, some common disadvantages of surveys were seen in this study including poor response rates, ambiguity in answers and possible misinterpretation of free text responses (Oppenheim, 2000), reducing the generalisability of the findings. The relatively small number of non-specialist SC responses meant meaningful comparison could not be made to specialist responses. Furthermore, many questions were at risk of non-response bias where a 100% response rate was not achieved.

Surveys provide a snapshot of information about what current behaviours are but usually provide little data about why this is the case (Mathers et al., 2009). The responses in this study evoke multiple follow-on questions, many of which would benefit from a qualitative approach to explore in detail.

Responses between SCs and OTs have been compared by identifying repeated topics using simple categorisation. The process was not standardised and is therefore at risk of subjective interpretation (Elo and Kyngäs, 2008). OTs and SCs were not paired so direct comparisons between responses must be interpreted with caution.

### Generalisability

Although the findings align with previous studies (Hodge et al., 2015; Onabolu et al., 2018) in recognising the contribution of OTs to efficiency and workforce enhancement, the generalisability is limited due to poor response rates, non-response bias, and the lack of pairing between SCs and OTs. The emergence of remote supervision is novel in the literature, although its clinical effectiveness compared to conventional supervision remains unexplored.

### Implications for clinical practice

The study highlights the importance of clear role definition and supervision structures between SCs and OTs, and the possible need to reconsider OT remuneration to reflect their clinical contribution. Some OTs reported undertaking, and some SCs reported prescribing, clinical procedures that are outside the defined OT scope. This study did not examine reasons for OTs working, or being prescribed to work, outside of their scope of practice. Possible explanations include ignorance of, or intentional disregard for, regulations. A minority of SCs (25%) and a larger group of OTs (68%) wanted an expansion in the permitted scope of practice of OTs, with comments left by some OTs expressing a desire for more autonomy. This should at least be reflected on during the development stage of any update to the GDC’s scope of practice guidance (General Dental Council, 2023b).

### Implications for research

Although SCs and OTs were not paired in this survey, it would be interesting to explore any differences in perceptions between OTs and SCs who work together. It is not known what effect the alignment of goals and priorities between SCs and OTs would have on delivery of orthodontic care. Organisational support theory research has shown that greater levels of teamwork led to improved job satisfaction in the NHS, which in turn positively impacted patient satisfaction (Ogbonnaya et al., 2018). Further research in this area could explore the potential for increased workforce and patient satisfaction through coordination of the aims of OTs and SCs.

Further exploration into remote supervision models, particularly the use of AI monitoring systems, should critically assess their safety, effectiveness and clinical efficiency within orthodontics.

## Conclusions

1. There were differences in the perceived benefits and challenges of co-delivered orthodontic treatment. OTs felt SCs should be more readily available and should improve communication, and that OTs should have more autonomy. SCs instead commented on preferring more time to supervise and provide prescriptions.
2. OTs reported improved patient satisfaction as the main consequence of their utilisation, whereas SCs described improved clinical efficiency.

## Supplemental Material

sj-pdf-1-joo-10.1177_14653125251391368 – Supplemental material for Co-delivery of orthodontic treatment: Perceptions of supervising clinicians and orthodontic therapists Supplemental material, sj-pdf-1-joo-10.1177_14653125251391368 for Co-delivery of orthodontic treatment: Perceptions of supervising clinicians and orthodontic therapists by Jonathan D Shelswell, Louise A Belfield, Simon J Littlewood and Sophy K Barber in Journal of Orthodontics
