# Supplementary material for: Co-delivery of orthodontic treatment: Perceptions of supervising clinicians and orthodontic therapists
Source: J Orthod. 2025 Nov 15;52(4):326–36. doi: 10.1177/14653125251391368 (PMC12664928; doi:10.1177/14653125251391368)
Supplement: sj-pdf-1-joo-10.1177_14653125251391368 – Supplemental material for Co-delivery of orthodontic treatment: Perceptions of supervising clinicians and orthodontic therapists [file sj-pdf-1-joo-10.1177_14653125251391368.pdf]

About this Questionnaire

This study aims to determine how treatment is currently delivered between orthodontic therapists (OT) and orthodontists/supervising dentists working in the UK.

This will be determined by establishing:

- The working arrangement of OTs with orthodontists/supervising dentists.
- Clinician perceptions of their contribution to patient care.
- Barriers and enablers to effective patient care between OTs and orthodontists/supervising dentists.

The questionnaire is being distributed nationally, and will be completed by both OTs and orthodontists/supervising dentists. For the purposes of this survey, 'supervising dentists' includes orthodontists, as well as dentists who regularly supervise OTs but do not have an MOrth qualification.

The questionnaire will take around 10-15 minutes to complete and is completely anonymous. It is divided into three sections:

1. What is the current structure of the supervising dentist and OT workforce?
2. How is treatment delivered between clinicians?
3. How do clinicians feel about the working relationship, and could it be more efficient?

This website follows strict data security standards (ISO27001) and is GDPR compliant and collects data over encrypted connections.

Eligibility

Please confirm that you are an orthodontic therapist or supervising orthodontist/supervising dentist **working in the United Kingdom**, and you have read the information about this study and give your consent to participate?

- Yes
- No

How to complete this questionnaire

The first part of this questionnaire will ask about your *main* place of work.

We recognise that you may work in multiple clinical settings. Please answer the questionnaire from the perspective of your main clinical role.

At the end, there will be an option to complete the questionnaire from the perspective of another clinical role. This is not mandatory, and only necessary if your responses would be largely different.

Main Clinical Role

What is your role at your *main* place of work? If you have multiple places of work, there will be an option at the end of the survey to repeat the questions from a different working perspective.

About You (OTs)

Where in the UK are you based?

- England
- Northern Ireland
- Scotland
- Wales

Do you have any other dental registrations, e.g., dental hygienist, dental therapist?

- [ ] Dental Nurse
- [ ] Dental Nurse with Additional Skills
- [ ] Dental Technician
- [ ] Clinical Dental Technician
- [ ] Dental Hygienist
- [ ] Dental Therapist

When and where did you complete your Diploma in Orthodontic Therapy?

As an approximate percentage, how is your week divided between primary and secondary care?

Primary care:

Secondary care:

Including you, how many orthodontic therapists work in your clinical setting?

Part time:

Full time:

How many orthodontists work in your clinical setting?

Part time:

Full time:

How many dental chairs in your clinical setting are typically in use for orthodontics?

What approximate percentage of orthodontics do you carry out on:

an NHS basis?

a private basis?

Do you feel your salary appropriately reflects your contribution to patient care?

- ○ Yes
- ○ No
- ○ Other

If you selected No or Other, please feel free to add comments:

Are you employed or self-employed?

- ☐ Employed
- ☐ Self-employed

If you feel comfortable to do so, please write your salary here. This question is not mandatory, and all responses are confidential.

How is treatment delivered in your practice? (OTs)

What is the standard length (in minutes) of your appointment times for the following:

Records

Treatment Planning/Consent

Placement of dual-arch fixed appliances

Adjusting fixed appliances

Removal of dual-arch fixed appliances

Aligner check (without attachment placement or interproximal reduction)

Placement of aligner attachments

How frequently does the supervising orthodontist see patients?

Are you provided with an overall treatment plan in the patient notes?

- Yes
- No
- Sometimes
- Varies between supervisors

Are you provided with a visit-by-visit treatment prescription?

- Yes - verbally
- Yes - written in the patient notes
- No

If yes, how often is this updated?

Does the visit-by-visit treatment prescription include:

- Individual recall intervals with supervising orthodontist
- Date/stage of treatment to reassess with supervising orthodontist
- Details regarding desired appliance mechanics
- None of the above
- Other

If you selected Other, please specify:

Do you have a standard operating procedure for orthodontic appliances in the absence of a supervisor?

If yes, please provide details:

Does the same OT complete the same patient's treatment from start to finish?

- Yes - always
- Yes - mostly
- Sometimes
- No

For the next question, please choose one of the following options:

- **Supervised** = under prescription with the orthodontist *available* to supervise.
- **Not supervised** = under prescription with the orthodontist ***not*** *available* to supervise that day.
- **Not done** = OT does not complete this procedure, but other clinicians do within your clinical setting.
- **N/A** = clinical procedure not undertaken by anyone in your clinical setting, e.g. aligners.

Which of the following clinical procedures do you carry out?

Please don't select more than 1 answer(s) per row.

	Supervised	Not supervised	Not done	N/A
IOTN screening				
Impressions/intra-oral scanning				
Pour, cast, and trim study models				
Bite registration				
Clinical photography				
Facebow records				
OHI, diet advice				
Taking radiographs				
Consent appointments				
Fitting separators				
Fitting bands				
Fitting headgear				
Fitting functional appliances				
Fitting passive removable appliances				
Activating components on removable appliances				

Bonding brackets	☐	☐	☐	☐
Changing elastomeric and ligature ties	☐	☐	☐	☐
Inserting archwires	☐	☐	☐	☐
Making changes to arch wires, e.g., expansion, bends, curves	☐	☐	☐	☐
Placing TADs	☐	☐	☐	☐
Utilising TADs	☐	☐	☐	☐
Removing TADs	☐	☐	☐	☐
Debonding	☐	☐	☐	☐
Cement/resin removal	☐	☐	☐	☐
Fitting retainers	☐	☐	☐	☐
Fitting fixed/bonded retainers	☐	☐	☐	☐
Interproximal reduction	☐	☐	☐	☐
Supra-gingival scaling	☐	☐	☐	☐
Sub-gingival scaling	☐	☐	☐	☐
Aligner review	☐	☐	☐	☐
Composite attachment placement	☐	☐	☐	☐
Teeth whitening	☐	☐	☐	☐
Emergency appointments	☐	☐	☐	☐

Do you feel your skills are being utilised appropriately?

If you selected No or Other, please specify:

Are there any other clinical procedures that you think you should be included in your scope of practice?

- ○ Yes
- ○ No

If you selected Yes, please specify: *Optional*

Do you wish you were able to make more decisions about treatment?

- ○ Yes
- ○ No

If you selected Yes, please specify: *Optional*

Are there any procedures that are only performed by the orthodontist?

- ☐ TADs
- ☐ IPR
- ☐ Archwire expansion
- ☐ IOTN scoring
- ☐ NP assessment
- ☐ Other

If you selected Other, please specify:

What is the **average** number of clinicians (including OTs and dentists) supervised by your supervising orthodontist?

What is the **maximum** number of clinicians (including OTs and dentists) supervised by your supervising orthodontist?

What do you feel is a sensible number of clinicians that should be supervised by a single dentist who is treating their own patients at the same time?

What do you feel is a sensible number of clinicians that should be supervised by a single dentist who is *not* treating their own patients at the same time?

Does your supervising dentist ever supervise remotely?

- Yes
- Sometimes
- No

If you selected Yes or Sometimes, please specify:

Does your supervising orthodontist perform any separate clinical activity whilst supervising?

- Yes - clinical admin
- Yes - treating/assessing patients
- No

How do you feel about your working relationship? (OTs)

How do you feel about your own contribution to the quality of patient care and treatment efficiency? E.g., treatment outcome, patient satisfaction, number of visits, appointment availability.

What do you feel works well with your working relationship with your supervising orthodontist(s)? E.g., communication, supervision.

What do you feel could be improved to maximise the working relationship?

About You (Orthodontists, Supervising Dentists and Orthodontic Trainees)

Where in the UK are you based?

- England
- Northern Ireland
- Scotland
- Wales

If you are an orthodontist, when did you complete your specialist training/CCST?

As an approximate percentage, how is your week divided between primary and secondary care?

Primary care:

Secondary care:

How many orthodontic therapists work in your clinical setting? Please include OTs you do not personally supervise.

Part time:

Full time:

Including you, how many orthodontists work in your clinical setting?

Part time:

Full time:

How many dental chairs in your clinical setting are typically in use for orthodontics?

What approximate percentage of their workload do the orthodontic therapists you work with carry out on:

an NHS basis?

a private basis?

How is treatment delivered in your practice? (Orthodontists, Supervising Dentists and Orthodontic Trainees)

The first questions on this page ask about your own clinical practice.

What is the standard length (in minutes) of your own appointment times for the following:

New patient assessment (clinical assessment only)

Records

Treatment planning/consent

Placement of dual-arch fixed appliances

Adjusting fixed appliances

Removal of dual-arch fixed appliances

Aligner check (without attachment placement or interproximal reduction)

Placement of aligner attachments

The next questions on this page ask about your supervision.

As the supervising dentist, how frequently do you see patients seen by your OT?

Do you provide an overall treatment plan in the patient notes?

- Yes
- No

Do you provide a visit-by-visit treatment prescription?

- Yes - verbally
- Yes - written in the patient notes
- No

If yes, how often is this updated?

Does the visit-by-visit treatment prescription include:

- Individual recall intervals with supervising orthodontist
- Date/stage of treatment to reassess with supervising orthodontist
- Details regarding desired appliance mechanics
- None of the above
- Other

If you selected Other, please specify:

Do you have a standard operating procedure for orthodontic appliances in the absence of a supervisor?

If yes, please provide details:

Does the same OT complete the same patient's treatment from start to finish?

- Yes - always
- Yes - mostly
- No

For the next question, please choose one of the following options:

- **Supervised** = under prescription with the orthodontist *available* to supervise.
- **Not supervised** = under prescription with the orthodontist ***not*** *available* to supervise that day.
- **Not done** = OT does not complete this procedure, but other clinicians do within your clinical setting.
- **N/A** = clinical procedure not undertaken by anyone in your clinical setting, e.g. aligners.

Which of the following clinical procedures does the OT you supervise carry out?

Please don't select more than 1 answer(s) per row.

	Supervised	Not supervised	Not done	N/A
IOTN screening				
Impressions/intra-oral scanning				
Pour, cast, and trim study models				
Bite registration				
Clinical photography				
Facebow records				
OHI, diet advice				
Taking radiographs				

Consent appointments	☐	☐	☐	☐
Fitting separators	☐	☐	☐	☐
Fitting bands	☐	☐	☐	☐
Fitting headgear	☐	☐	☐	☐
Fitting functional appliances	☐	☐	☐	☐
Fitting passive removable appliances	☐	☐	☐	☐
Activating components on removable appliances	☐	☐	☐	☐
Bonding brackets	☐	☐	☐	☐
Changing elastomeric and ligature ties	☐	☐	☐	☐
Inserting archwires	☐	☐	☐	☐
Making changes to arch wires, e.g., expansion, bends, curves	☐	☐	☐	☐
Placing TADs	☐	☐	☐	☐
Utilising TADs	☐	☐	☐	☐
Removing TADs	☐	☐	☐	☐
Debonding	☐	☐	☐	☐
Cement/resin removal	☐	☐	☐	☐
Fitting retainers	☐	☐	☐	☐
Fitting fixed/bonded retainers	☐	☐	☐	☐
Interproximal reduction	☐	☐	☐	☐
Supra-gingival scaling	☐	☐	☐	☐
Sub-gingival scaling	☐	☐	☐	☐
Aligner review	☐	☐	☐	☐
Composite attachment placement	☐	☐	☐	☐
Teeth whitening	☐	☐	☐	☐
Emergency appointments	☐	☐	☐	☐

Do you feel the skills of OTs you work with are being utilised appropriately?

If you selected No or Other, please specify:

Are there any other clinical skills that you think OTs should have?

- Yes
- No

If you selected Yes, please specify: *Optional*

Do you wish OTs were able to make more decisions about treatment?

- Yes
- No

If you selected Yes, please specify: *Optional*

Are there any procedures that only you perform?

- TADs
- IPR
- Archwire expansion
- IOTN scoring
- NP assessment
- Other

If you selected Other, please specify:

What is the **average** number of clinicians (including OTs and dentists) you supervise at any one time?

What is the **maximum** number of clinicians (including OTs and dentists) you would supervise at any one time?

What do you feel is a sensible number of clinicians that should be supervised by a single dentist who is treating their own patients at the same time?

What do you feel is a sensible number of clinicians that should be supervised by a single dentist who is *not* treating their own patients at the same time?

Do you ever supervise remotely?

- Yes
- Sometimes
- No

If you selected Yes or Sometimes, please specify:

Do you perform any separate clinical activity whilst supervising?

- Yes - clinical admin
- Yes - treating/assessing patients
- No

How do you feel about your working relationship? (Orthodontists, Supervising Dentists and Orthodontic Trainees)

What positive or negative effect does the contribution of OTs you work with have on the quality of patient care and treatment efficiency? E.g., treatment outcome, quality of finish, patient satisfaction, number of visits, appointment availability.

What effect does working with an OT have on your own clinical practice? E.g., number of patients seen, more/less stressful.

What do you feel works well with your working relationship with your orthodontic therapist(s)? E.g., communication, supervision.

What do you feel could be improved to maximise the working relationship?

Additional role?

Do you wish to repeat the questionnaire from another working perspective, if you feel your responses would be notably different? ✱ *Required*
